# Impact of red blood cell transfusion (RBCT) on outcome among septic patients admitted into intensive care unit (ICU), a systematic review and meta-analysis

**DOI:** 10.1186/2197-425X-3-S1-A641

**Published:** 2015-10-01

**Authors:** C Dupuis, R Sonneville, W Essaied, S Ruckly, L Bouadma, JF Timsit

**Affiliations:** INSERM UMR 1137, Paris, France; CHU Bichat, Paris, France

## Introduction

RBCT is still a subject of debate with 2 main questions: what is the optimal threshold? and does transfusion impact the outcome?. Concerning septic patients, a threshold of 10 g/dL was for many years the objective during the first hours of septic shock and since the last surviving sepsis campaign, a threshold of 7 g/dL for stabilized septic patients is proposed. Nevertheless, the evidence base dealing with septic patients remains poor and cohort studies bring divergent results.

## Objectives

The aim of this study was to achieve a systematic review and meta-analysis on the impact of RBCT among septic ICU adults.

## Methods

DATA SOURCES MEDLINE, EMBASE, Web of Science Core Collection, Cochrane Central Register of Controlled Trials, Cochrane Database of Systematic Reviews and Clinical Trials.gov were searched through January 01, 2015.

## Study Selection

We included randomized clinical trials (RCT) comparing restrictive versus liberal RBCT strategies and cohort studies assessing the impact of RBCT on septic ICU adults.

## Data Extraction and Synthesis

Two RCT and 34 cohort studies were included into the systematic review and 30 into the meta-analysis. Death, nosocomial infection (NI) and acute lung injury (ALI) were the main outcome measures. Der Simonian and Laird random-effects models were used to report pooled odd ratios (OR). Sub group analysis and meta-regressions were achieved in order to explore heterogeneity of the data.

## Results

The 2 RCT were the study of Hebert ([1]) with only 4.9% of septic patients and the study of Holst ([2]). In both studies, the rates of outcome measure were not different for restrictive strategy.

Eighteen cohort studies and 10992 patients were included into the meta-analysis dealing with death. RBCT improved mortality (overall pooled OR was 1.33[1.13-1.57]; I²=77%, p < 0.001). But in the sub group analysis of the studies specific of septic patients (n=7), mortality was unaffected by RBCT (pooled OR was 1.10[0.75, 1.60]; I²=57%, p = 0.03). The heterogeneity could be explained by the statistical methods (sub group analysis of the 11 studies with multivariate analyses: pooled OR=1.35[1.18-1.54]; I²=44%, p = 0.06)). A better outcome was found when pooling the 3 studies with Cox model (pooled HR = 0.70[0.49-0.98]; I²=89%, p < 0.001). Transfusion was associated with the occurrence of NI (8 studies, pooled OR: 1.94[1.35-2.79]; I²=92%, p < 0.001) and the occurrence of ALI (8 studies: 1.98[1.41-2.78]; I²=7, p < 0.001).

## Conclusions

When analyzing all the studies, RBCT is associated with death, NI and ALI. In septic patients however, neither the only available RCT nor the meta-analysis of cohort studies showed an effect of RBC strategy on mortality. All those results must be interpreted with caution given the heterogeneity which could be explained in part by the percentage of septic patients and the statistical methods.
